# Multidisciplinary Intensive Care Management of Polyneuropathy, Organomegaly, Endocrinopathy, Monoclonal Protein, and Skin Changes (POEMS) Syndrome: A Case Report

**DOI:** 10.7759/cureus.95290

**Published:** 2025-10-24

**Authors:** Zaza Aladashvili, Thalia B Rodriguez, Elene Zaalishvili, Luka Beridze, Yaroslav Buryk

**Affiliations:** 1 Internal Medicine, Tbilisi State Medical University, Tbilisi, GEO; 2 Medicine, Ross University School of Medicine, Miramar, USA; 3 Internal Medicine, Jackson Memorial Hospital, Miami, USA; 4 Internal Medicine, David Tvildiani Medical University, Tbilisi, GEO; 5 Internal Medicine, Petre Shotadze Tbilisi Medical Academy, Tbilisi, GEO; 6 Pulmonary and Critical Care, Jackson Memorial Hospital, Miami, USA

**Keywords:** acute kidney injury, hemodynamic instability, multiorgan dysfunction, nodular regenerative hyperplasia, poems syndrome, portal hypertension

## Abstract

We present the case of a 65-year-old man with long-standing polyneuropathy, organomegaly, endocrinopathy, monoclonal protein, and skin changes (POEMS) syndrome, who developed progressive multi-organ dysfunction requiring support in the intensive care unit (ICU). The patient’s course was complicated by nodular regenerative hyperplasia (NRH) contributing to non-cirrhotic portal hypertension and severe ascites, acute kidney injury (AKI), hemodynamic instability, and endocrine abnormalities. Management involved multidisciplinary coordination across critical care, hematology, nephrology, and endocrinology teams, with prophylactic antimicrobial therapy, careful hemodynamic optimization, and individualized nutritional and endocrine support. This case highlights the complexity of ICU management in POEMS syndrome, demonstrating the importance of early recognition, collaborative care, and tailored interventions in optimizing outcomes for critically ill patients.

## Introduction

POEMS syndrome is a rare multisystem disorder, named for its cardinal features: polyneuropathy, organomegaly, endocrinopathy, monoclonal protein, and skin changes [1,2]. It arises from an underlying plasma cell disorder, although its precise pathogenesis remains unclear [3,4]. Current evidence suggests that clonal or polyclonal plasma cells, often producing monoclonal lambda light chains, drive disease activity, partly through overproduction of vascular endothelial growth factor (VEGF) and other inflammatory cytokines [2]. The prevalence is estimated at 0.3 per 100,000 in Japan [3]. Median age at onset is 46 years (range: 10-81), with a male-to-female ratio of 2.23:1 in the Chinese population [1].

Diagnosis requires careful clinical evaluation supported by targeted investigations, including radiographic bone assessment, serum VEGF measurement, and bone marrow biopsy [3]. Delays in diagnosis are common, as presenting symptoms may be subtle or misattributed, particularly when neuropathy precedes other manifestations [5].

Treatment targets both the underlying plasma cell clone and symptom control [3]. Management decisions begin with determining bone marrow involvement and the number of sclerotic bone lesions. Patients without marrow involvement and with fewer than two lesions are candidates for localized radiation, whereas those with marrow infiltration require systemic chemotherapy, commonly lenalidomide, dexamethasone, or cyclophosphamide [3,5]. Supportive care includes antithrombotic and antimicrobial prophylaxis, along with psychological support [5].

Despite its chronic course, median survival approaches 165 months, independent of initial presentation [2]. Most deaths result from cardiorespiratory failure or infection, and renal failure-related mortality is often associated with concurrent ascites and capillary leak-like syndrome [2].

Here, we present a patient with POEMS syndrome who developed multi-organ dysfunction requiring intensive care unit (ICU) support. The case illustrates the complexity of management and the importance of a multidisciplinary approach in this rare condition.

## Case presentation

A 65-year-old man with a history of POEMS syndrome (diagnosed in 2005), presenting with polyneuropathy, organomegaly, endocrinopathies (androgen deficiency and hypothyroidism), monoclonal gammopathy, and skin changes, vitamin D deficiency, osteopenia, umbilical hernia, and recently diagnosed non-cirrhotic portal hypertension secondary to nodular regenerative hyperplasia (NRH) four weeks prior to presentation, presented with one week of progressive generalized weakness, worsening after a transjugular liver biopsy performed four days prior. Over the preceding 2-3 weeks, he had developed increasing abdominal distension, lower extremity edema, and exertional dyspnea.

His POEMS syndrome had been treated with left hip radiation in 2005 and systemic therapy with lenalidomide, cyclophosphamide, and dexamethasone in 2020. The recent liver biopsy confirmed NRH with a hepatic venous pressure gradient of 12 mmHg. Home medications included furosemide, spironolactone, levothyroxine, atorvastatin, calcium carbonate, and ergocalciferol.

On arrival, he was afebrile, hypotensive (92/59 mmHg), and bradycardic (55 bpm), with oxygen saturation of 99% on room air. Physical examination revealed abdominal distension with a positive fluid wave and an umbilical hernia; cardiopulmonary and neurological examinations were unremarkable. Table 1 presents the patient’s vital signs and selected laboratory parameters at admission, their lowest or highest recorded values during hospitalization, the most recent available measurements, and the corresponding reference ranges.

**Table 1:** Summary of key vital signs and laboratory findings during hospitalization

Parameter	On admission	Lowest/highest recorded	Recent/last available	Reference range
Temperature (°C)	36.7	35.5-36.7	36	36.1-37.2
Heart rate (bpm)	55	51	57	60-100
Blood pressure (mmHg)	92/59	90/55	93/57	100-140/60-90
Respiratory rate (breaths/min)	14	14-16	16	12-20
SpO₂ (%)	99	99	100	≥95
Blood urea nitrogen (mg/dL)	137	-	-	8-20
Creatinine (mg/dL)	3	2.3-3.0	2.3	0.6-1.3
Potassium (mmol/L)	7.4	4.4-7.4	4.4	3.5-5.0
Bicarbonate (mmol/L)	17	-	-	22-28
Platelets (×10³/µL)	104	40-104	40	150-450
Hemoglobin (g/dL)	-	>7 (maintained)	-	13.5-17.5
Hepatic venous pressure gradient (mmHg)	12	-	-	-
Ascitic fluid (mL)	3500 (initial)	4.7 L (total)	-	-

Initial laboratory results demonstrated stage 3 acute kidney injury (AKI) (BUN 137 mg/dL, creatinine 3.0 mg/dL; baseline ~1.2 mg/dL), severe hyperkalemia (7.4 mmol/L) with electrocardiographic changes, non-anion gap metabolic acidosis, and thrombocytopenia (104 × 10³/µL, down from 162 × 10³/µL one week earlier). Liver function tests were near baseline. An initial diagnostic paracentesis at admission yielded 3.5 L of ascitic fluid, negative for spontaneous bacterial peritonitis. Infectious workup, including hepatitis serologies, was negative.

He was admitted to the intermediate care unit for emergent hyperkalemia management with intravenous insulin-dextrose, potassium binders, and dietary potassium restriction. Nephrology attributed the AKI to multifactorial causes, including hypoperfusion, possible acute tubular necrosis, and hepatorenal physiology; diuretics were discontinued, and albumin infusions were initiated. When hypotension progressed, broad-spectrum antibiotics were started for suspected sepsis, and vasopressor support with norepinephrine and midodrine was initiated. Octreotide was added for possible hepatorenal syndrome. Later during hospitalization, hepatology performed a large-volume therapeutic paracentesis (4.7 L removed) for refractory ascites. They also considered transjugular intrahepatic portosystemic shunt (TIPS) if diuretic therapy remained intolerable. The complete list of inpatient medications and their indications is provided in Table 2.

**Table 2:** Inpatient medications administered during hospitalization and their indications AKI: acute kidney injury, GI: gastrointestinal, IV: intravenous, PO: oral, SC: subcutaneous, SubQ: subcutaneous.

Medication	Indication	Route
Norepinephrine	Vasopressor for hypotension/septic shock	IV infusion
Midodrine	Vasopressor support	PO
Albumin (25%)	Volume expansion in portal hypertension/AKI	IV
Octreotide	Hepatorenal syndrome management	IV/SC
Cefepime	Broad-spectrum antibiotic	IV
Vancomycin	Broad-spectrum antibiotic	IV
Lokelma	Hyperkalemia	PO
Citric acid-sodium citrate (Bicitra)	Metabolic acidosis	PO
Levothyroxine	Hypothyroidism	PO
Rosuvastatin	Hyperlipidemia	PO
Calcium carbonate	Hypocalcemia/osteopenia	PO
Ergocalciferol	Vitamin D deficiency	PO
Famotidine	GI prophylaxis	PO
Dextrose 50%	Hypoglycemia/hyperkalemia protocol	IV push
Glucagon	Severe hypoglycemia	SubQ
Ondansetron	Nausea	IV
Stress-dose steroids	Suspected adrenal insufficiency in shock	IV

Cardiology evaluation of telemetry abnormalities revealed sinus bradycardia, accelerated idioventricular rhythm, and premature ventricular contractions, attributed to electrolyte disturbances; no direct intervention was required. Hematology assessed the thrombocytopenia as likely multifactorial, related to portal hypertension-associated splenic sequestration, sepsis, and medication effects. Palliative care discussions confirmed the patient’s preference to remain full code with maximal medical support. At the last follow-up, he remained in critical care on vasopressors and albumin infusions, with partial improvement in renal function but persistent hemodynamic instability.

The patient remained hospitalized for a total of 37 days, during which his condition deteriorated, necessitating mechanical ventilation and continuous vasopressor support. At the end of the observation period, he was critically ill with ongoing multi-organ dysfunction.

## Discussion

What makes this case particularly noteworthy is the rare and complex interplay between long-standing POEMS syndrome and the later development of NRH, which resulted in non-cirrhotic portal hypertension and AKI. This combination added a significant burden to an already multisystem disease, highlighting the diagnostic and therapeutic challenges of managing rare disorders when new, uncommon complications emerge.

Liver involvement in POEMS syndrome is uncommon, and even more rarely has it been associated with NRH. To date, only three reported cases describe idiopathic portal hypertension in POEMS patients, and none of them mention NRH [6]. NRH typically results from microvascular injury or disrupted blood flow within the portal venous system. Localized portal hypoperfusion leads to hepatocyte atrophy, while neighboring areas with relatively preserved blood flow undergo compensatory hypertrophy and nodule formation. Common underlying causes of NRH include autoimmune diseases, hematologic conditions, certain medications (such as azathioprine and chemotherapies), hypercoagulable states, and vasculopathies. The patient had prior exposure to lenalidomide, cyclophosphamide, and dexamethasone, which are chemotherapeutic agents that may contribute to microvascular injury and could have played a role in the development of NRH.

POEMS syndrome, on the other hand, is characterized by significantly elevated VEGF levels, along with increased inflammatory cytokines such as IL‑1β, IL‑6, and TNF-α. VEGF promotes angiogenesis and increases vascular permeability, leading to hypervascularization across multiple organ systems. While VEGF in POEMS syndrome clearly alters vascular architecture and permeability, NRH arises specifically from microvascular occlusion or uneven blood flow that causes localized ischemia and regenerative nodule formation, not simply from vascular leakage or proliferation. Interestingly, one case report (unrelated to POEMS) linked bevacizumab, a VEGF-blocking antibody, and oxaliplatin treatment with the development of portal vein thrombosis and subsequent NRH [7]. Though not direct evidence of VEGF causing NRH, it suggests that disturbances in VEGF signaling may contribute to vascular injury and NRH pathogenesis.

Hepatomegaly and splenomegaly are relatively common in POEMS syndrome, reported in approximately 50%-78% of patients [8]. Additionally, around 80% experience extravascular volume overload, manifesting as ascites, peripheral edema, and pleural or pericardial effusions [9]. In some cases, ascites can be a prominent and even the initial feature. For example, one reported case, a confirmed diagnosis of POEMS syndrome, described ascites developing a year after the onset of neuropathy, with no underlying cardiac or liver disease, highlighting POEMS as a rare but important cause of refractory ascites [10]. In our case, the combination of NRH, POEMS syndrome, and non-cirrhotic portal hypertension significantly complicated both diagnosis and management. Identifying the primary driver of portal hypertension and fluid accumulation proved particularly challenging.

The patient also developed stage 3 AKI, likely due to multiple contributing factors: volume depletion, hypotension, and underlying portal hypertension. These factors suggest a combination of pre-renal azotemia, acute tubular necrosis, and hepatorenal physiology. Recent large-volume paracentesis, use of diuretics, and bradycardia, likely secondary to electrolyte abnormalities, and overall hemodynamic instability further compromised renal perfusion. Management required discontinuation of diuretics, volume repletion with albumin, and vasopressor support. Octreotide was initiated empirically for suspected hepatorenal syndrome (HRS), although its effectiveness in non-cirrhotic portal hypertension remains unclear.

Importantly, POEMS syndrome itself has been associated with kidney disease. In the largest review of renal involvement in POEMS syndrome, 52 cases were analyzed, with kidney biopsies available for 22 patients [11]. Approximately half had serum creatinine levels above 1.5 mg/dL, and 10% required dialysis. Pathologic findings were primarily glomerular, including glomerular enlargement, mesangiolysis, cellular proliferation, and prominent endothelial swelling. In our patient, a renal biopsy was considered but ultimately deferred as renal function improved with supportive care. Whether the patient’s renal injury was exacerbated by direct renal involvement from POEMS or was entirely secondary to hepatic dysfunction and hemodynamic instability remains uncertain.

POEMS syndrome is well recognized for its multisystem involvement, affecting the peripheral nervous system, endocrine organs, skin, and vasculature, among others. However, it also raises the question of whether the syndrome may predispose patients to additional complications or comorbid conditions beyond its classical manifestations. The chronic inflammatory state, elevated cytokine levels (particularly VEGF), and altered immune regulation may render patients more vulnerable to secondary organ dysfunction or failure. This case underscores the importance of early recognition, close monitoring, and proactive, multidisciplinary management in preventing or mitigating multi-organ involvement. Coordinated care among neurology, hematology, nephrology, hepatology, and critical care teams is essential to address the complex and evolving clinical course of POEMS syndrome.

## Conclusions

This case illustrates the challenges of managing a patient with POEMS syndrome complicated by multi-organ dysfunction, including non-cirrhotic portal hypertension secondary to NRH and AKI. The patient’s clinical course involved progressive hemodynamic instability, fluid overload, and endocrine abnormalities, highlighting the dynamic interplay of multiple organ systems in this rare disorder. Careful evaluation, close monitoring, and coordinated interventions from various medical teams were essential in addressing the patient’s complex needs.

The management of such patients requires individualized therapeutic strategies, including careful fluid and electrolyte balance, targeted pharmacologic interventions, and procedural support when indicated, such as large-volume paracentesis. This case demonstrates how timely recognition of evolving complications, proactive supportive care, and multidisciplinary collaboration can influence outcomes, even in patients with severe presentations.
